# A Cytopathic Effect-Based Tissue Culture Method for HCoV-OC43 Titration Using TMPRSS2-Expressing VeroE6 Cells

**DOI:** 10.1128/mSphere.00159-21

**Published:** 2021-05-12

**Authors:** Ryohei Hirose, Naoto Watanabe, Risa Bandou, Takuma Yoshida, Tomo Daidoji, Yuji Naito, Yoshito Itoh, Takaaki Nakaya

**Affiliations:** a Department of Infectious Diseases, Graduate School of Medical Science, Kyoto Prefectural University of Medicine, Kyoto, Japan; b Department of Molecular Gastroenterology and Hepatology, Graduate School of Medical Science, Kyoto Prefectural University of Medicine, Kyoto, Japan; c Department of Forensics Medicine, Graduate School of Medical Science, Kyoto Prefectural University of Medicine, Kyoto, Japan; Icahn School of Medicine at Mount Sinai

**Keywords:** human coronavirus, HCoV-OC43, TMPRSS2, 50% tissue culture infectious dose assay, cytopathic effect, titration

## Abstract

Human coronavirus (HCoV)-OC43 rarely shows a cytopathic effect (CPE) after infection of various cell lines, and the indirect immunoperoxidase assay (IPA), a relatively complex procedure, has long been used as an alternative assay. Because HCoV-OC43 uses cell-surface transmembrane protease serine 2 (TMPRSS2) for cell entry, VeroE6 cells expressing TMPRSS2 may show a clear CPE after HCoV-OC43 infection. The aim of this study was to construct a 50% tissue culture infectious dose (TCID_50_) assay for HCoV-OC43 based on CPE evaluation using VeroE6/TMPRSS2 cells. VeroE6/TMPRSS2 cells showed clear CPEs 3 to 4 days after low-titer HCoV-OC43 infection. Evaluation of viral kinetics indicated that the viral titer in the culture supernatant of VeroE6/TMPRSS2 cells in the early stages of infection was higher than that of other cells. In comparison, between the CPE-based and the IPA-based (i.e., the reference titer) methods, the titer measured with CPE evaluation 4 to 5 days after infection using VeroE6/TMPRSS2 cells showed a much smaller difference from the reference titer than that measured using other cells. Thus, the TCID_50_ assay using CPE evaluation with VeroE6/TMPRSS2 cells provides the correct titer value and will greatly contribute to future research on HCoV-OC43.

**IMPORTANCE** HCoV-OC43 rarely shows a cytopathic effect (CPE) in infected cell lines, and thus the plaque and TCID_50_ assays by CPE observation are not applicable for titration; the indirect immunoperoxidase assay (IPA) is used instead. However, the IPA is relatively complex, time-consuming, costly, and not suitable for simultaneous titration of many samples. We developed a TCID_50_ assay using CPE evaluation with TMPRSS2-expressing VeroE6/TMPRSS2 cells that provides the same accuracy as the conventional IPA-based viral titration and does not require any staining procedures using antibodies or substrates. This titration method will greatly contribute to future research on HCoV-OC43 by allowing simple, low-cost, and accurate titration of this virus.

## Abstract (precis)

HCoV-OC43 rarely shows a cytopathic effect (CPE) in infected cell lines, and thus the plaque and TCID_50_ assays by CPE observation are not applicable for titration; the indirect immunoperoxidase assay (IPA) is used instead. However, the IPA is relatively complex, time-consuming, costly, and not suitable for simultaneous titration of many samples.

## INTRODUCTION

Human coronavirus (HCoV) causes the common cold in humans. Four HCoVs (229E, NL63, OC43, and HKU1) have been identified so far. HCoV-229E and HCoV-NL63 belong to the *Alphacoronavirus* genus and HCoV-OC43 and HCoV-HKU1 to the *Betacoronavirus* genus (1–3). Because HCoV-OC43 belongs to the same *Betacoronavirus* genus as Middle East respiratory syndrome coronavirus (MERS-CoV), severe acute respiratory syndrome coronavirus 1 (SARS-CoV-1), and SARS-CoV-2, it may be used in various studies in the future as a comparison target for these highly pathogenic coronaviruses.

Accurate and reproducible measurement of viral titer is the most fundamental and important aspect of virus research. Because HCoV-OC43 rarely shows a clear cytopathic effect (CPE) in infections of various cell lines, the classical plaque assay and the 50% tissue culture infectious dose (TCID_50_) assay by CPE observation are not applicable for titration (4, 5). Therefore, the indirect immunoperoxidase assay (IPA) has long been used as an alternative assay method. Specifically, virus detection by IPA 4 days after viral infection determines the viral titer, expressed as 50% tissue culture infectious dose (TCID_50_) (4–9). IPA has been widely used as a reliable method for titrating HCoV-OC43 in samples. However, the procedure for IPA is relatively complex, time-consuming, and costly and is not suitable for simultaneous titration of many samples. Additionally, a stable supply of suitable antibodies is essential for the assay. Therefore, although HCoV-OC43 is important as a comparison target for SARS-CoV-2, evaluations using HCoV-OC43 that require frequent virus titration, such as virus stability and disinfection effect evaluations, have not been actively performed (10–13). Establishing a simpler, faster, and cost-effective titration method for HCoV-OC43 is thus desirable.

Previous studies suggested that SARS-CoV-1 and SARS-CoV-2 are proteolytically activated by transmembrane protease serine 2 (TMPRSS2), and VeroE6/TMPRSS2 cells expressing TMPRSS2 are highly susceptible to these viruses (14–16). In fact, because VeroE6/TMPRSS2 cells show CPE within a few days even with low-titer SARS-CoV-2 infection, they have become widely used for titration of this virus (17, 18).

HCoV-OC43 enters cells via two distinct pathways as follows: the endosomal pathway, using cathepsins to activate the spike protein, and the cell-surface or early endosome pathway, using TMPRSS2. Previous studies suggested that HCoV-OC43 generally uses the cell-surface TMPRSS2 for cell entry and not endosomal cathepsins (19–21). Therefore, we hypothesized that VeroE6/TMPRSS2 cells are highly susceptible to HCoV-OC43 infection and may therefore show clear CPE.

Based on this hypothesis, we aimed to develop a TCID_50_ assay method for HCoV-OC43 with CPE observation using VeroE6/TMPRSS2 cells and compared it to the standard titer measurement with IPA using HCT-8 cells. The proposed method provided the correct titer value and will greatly contribute to future research on HCoV-OC43.

## RESULTS

### CPE evaluation.

**FIG 1:**
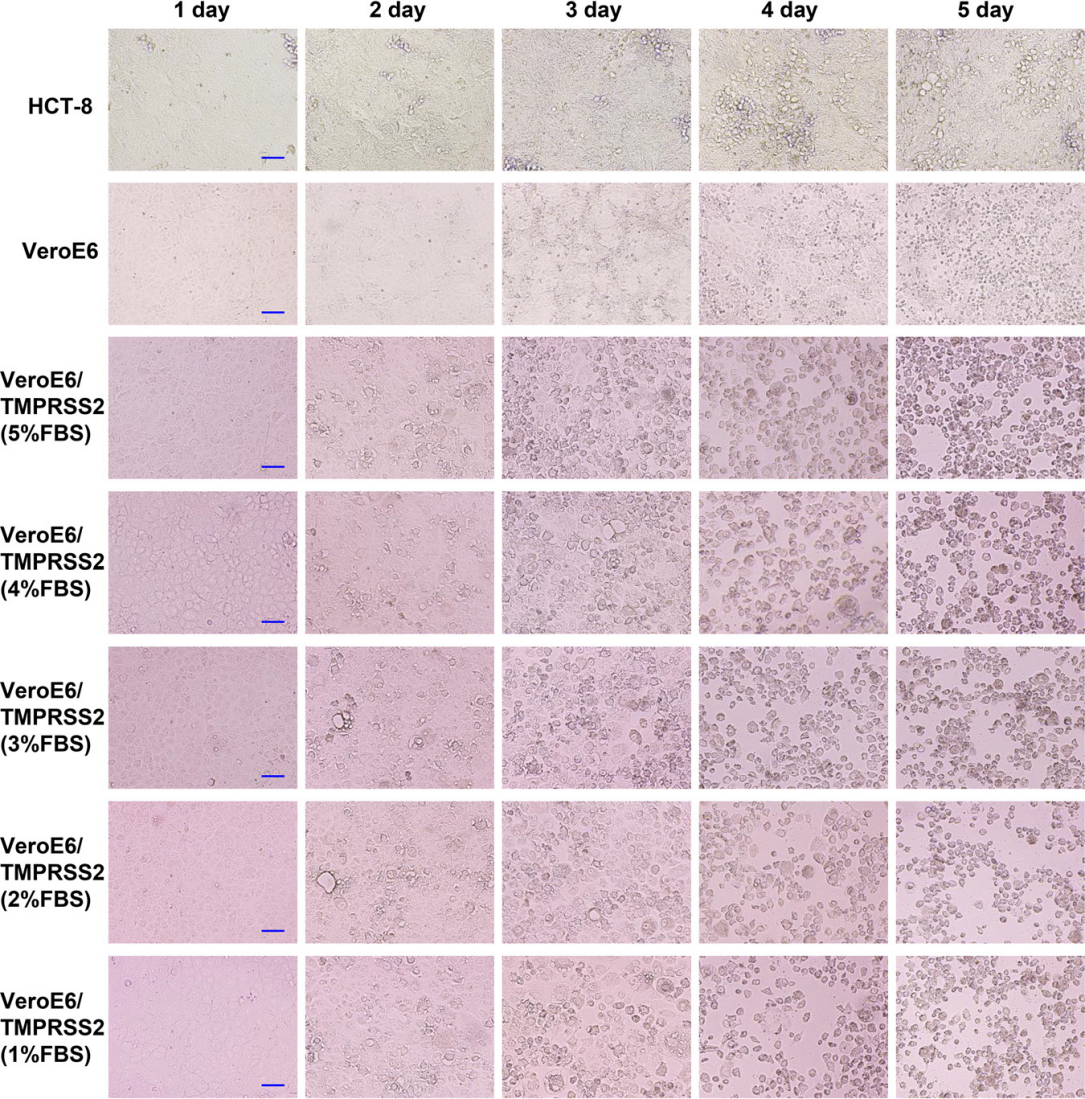
CPE evaluation after high-titer HCoV-OC43 infection. First, HCT-8, VeroE6, and VeroE6/TMPRSS2 cells were seeded in 96-well plates and infected with high-titer (2.0 × 10^5^ TCID_50_/ml) HCoV-OC43 samples, and their CPEs were evaluated from 1 to 5 days after infection. CPE was observed with an inverted light microscope (Olympus IX71; Olympus, Tokyo, Japan). VeroE6/TMPRSS2 cells were cultured in DMEM supplemented with 1%, 2%, 3%, 4%, or 5% fetal bovine serum after virus inoculation. Scale bar, 100 μm.

**FIG 2:**
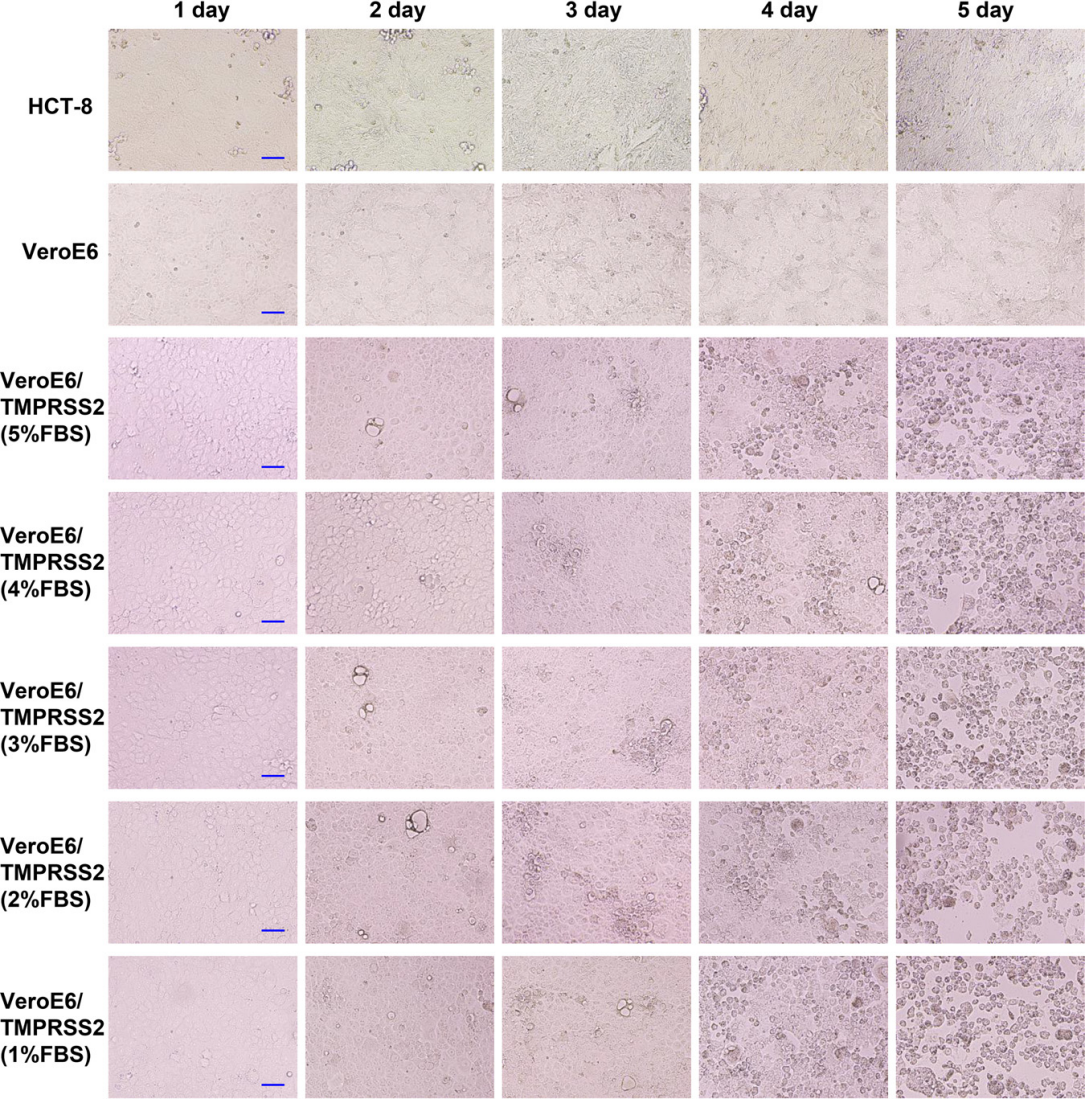
CPE evaluation after medium-titer HCoV-OC43 infection. First, HCT-8, VeroE6, and VeroE6/TMPRSS2 cells were seeded in 96-well plates and infected with medium-titer (2.0 × 10^3^ TCID_50_/ml) HCoV-OC43 samples, and their CPEs were evaluated from 1 to 5 days after infection. CPE was observed with an inverted light microscope (Olympus IX71; Olympus, Tokyo, Japan). VeroE6/TMPRSS2 cells were cultured in DMEM supplemented with 1%, 2%, 3%, 4%, or 5% fetal bovine serum after virus inoculation. Scale bar, 100 μm.

**FIG 3:**
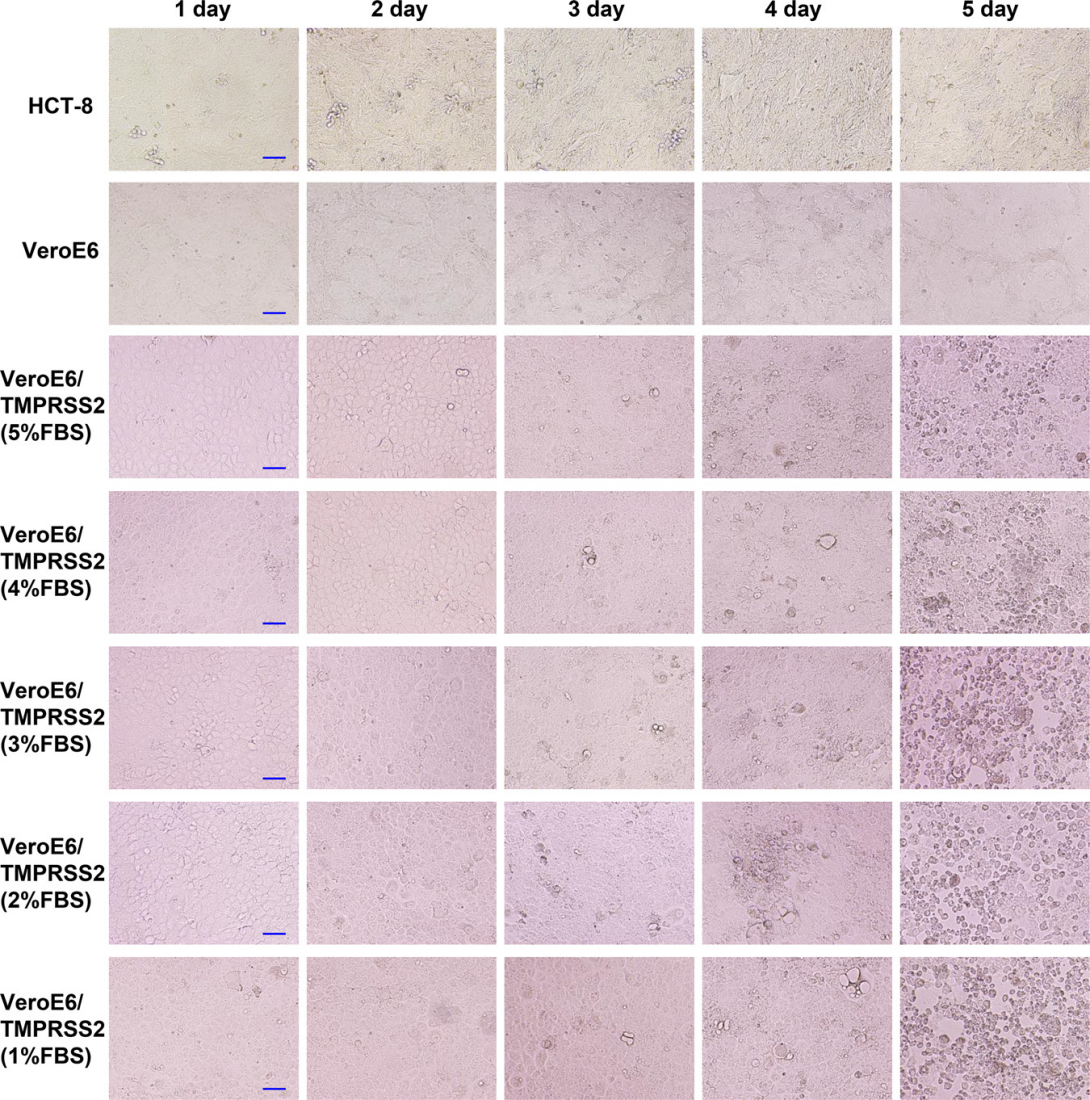
CPE evaluation after low-titer HCoV-OC43 infection. First, HCT-8, VeroE6, and VeroE6/TMPRSS2 cells were seeded in 96-well plates and infected with low-titer (2.0 × 10^1^ TCID_50_/ml) HCoV-OC43 samples, and their CPEs were evaluated from 1 to 5 days after infection. CPE was observed with an inverted light microscope (Olympus IX71; Olympus, Tokyo, Japan). VeroE6/TMPRSS2 cells were cultured in DMEM supplemented with 1%, 2%, 3%, 4%, or 5% fetal bovine serum after virus inoculation. Scale bar, 100 μm.

HCoV-OC43 sample solutions at high titer, medium titer, and low titer were used to infect HCT-8, Vero, and VeroE6/TMPRSS2 cells, and then the CPE was evaluated (Table 1 and Fig. 1, 2, and 3). In HCT-8 and VeroE6 cells, CPE was observed 4 days after infection when infected with high-titer HCoV-OC43, and no CPE was observed when infected with medium-titer or low-titer HCoV-OC43. In VeroE6/TMPRSS2 cells, CPE was observed 2 days after infection when infected with high-titer and medium-titer HCoV-OC43, and CPE was observed 4 days after infection when infected with low-titer HCoV-OC43. In addition, CPE tended to be observed slightly earlier when the fetal bovine serum (FBS) concentration in the cell culture medium was lowered.

**TABLE 1:** CPE evaluation of each cell line^*a*^

Cell line	Result according to titer (TCID_50_/ml) and day (1–5)														
	2.0 × 10^5^					2.0 × 10^3^					2.0 × 10^1^				
	1	2	3	4	5	1	2	3	4	5	1	2	3	4	5
HCT-8	−	−	+−	+	+	−	−	−	−	−	−	−	−	−	−
VeroE6	−	−	+−	+	+	−	−	−	−	−	−	−	−	−	−
VeroE6/TMPRSS2 (5% FBS)	−	+	+	++	++	−	+−	+−	+	++	−	−	+−	+	+
VeroE6/TMPRSS2 (4% FBS)	−	+	+	++	++	−	+−	+−	+	++	−	−	+−	+	+
VeroE6/TMPRSS2 (3% FBS)	−	+	+	++	++	−	+−	+	+	++	−	−	+−	+	++
VeroE6/TMPRSS2 (2% FBS)	−	+	+	++	++	−	+−	+	+	++	−	−	+−	+	++
VeroE6/TMPRSS2 (1% FBS)	−	+	+	++	++	−	+−	+	+	++	−	−	+−	+	++

a −, CPE not observed; +−, CPE slightly observed; +, CPE clearly observed; ++, CPE very clearly observed. See Fig. 1, 2, and 3 for micrographs of CPE under each condition.

### Evaluation of viral kinetics.

**FIG 4:**
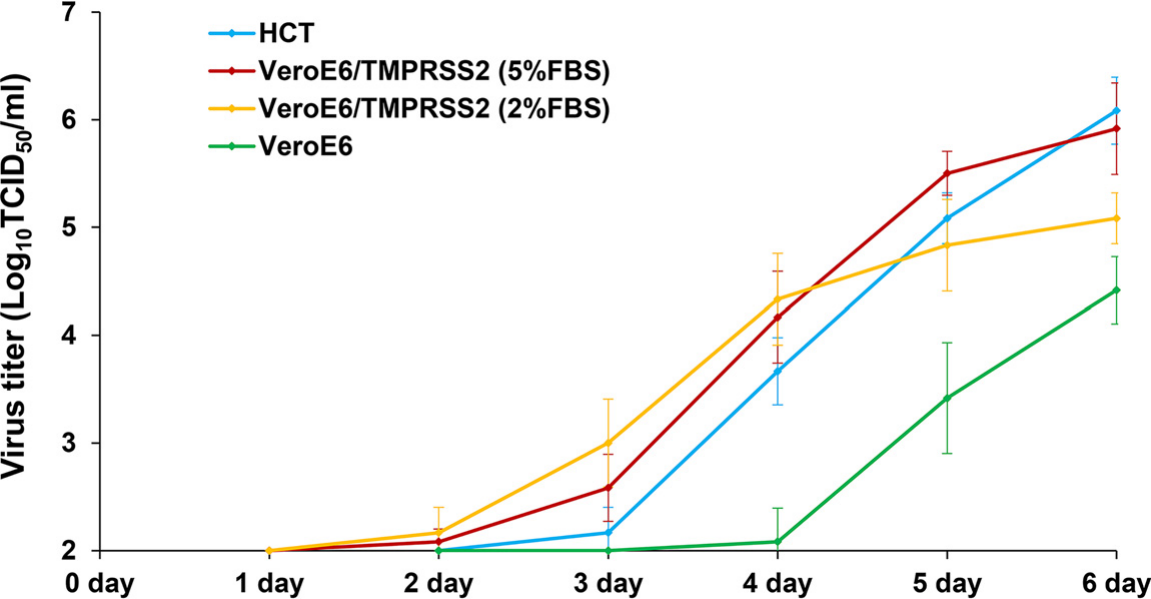
Viral kinetics of HCoV-OC43. HCT-8, VeroE6, and VeroE6/TMPRSS2 cells were seeded in 12-well plates and infected with a specific titer of HCoV-OC43 (1.0 × 10^2^ TCID_50_/ml). The viral titer in the culture supernatant of each well was measured from 1 to 6 days after infection. VeroE6/TMPRSS2 cells were cultured in DMEM supplemented with 2% or 5% fetal bovine serum after virus inoculation. For each measurement, three independent experiments were performed, and the results are expressed as mean ± standard error values.

The virus in the culture supernatant began to increase 2 days after infection of VeroE6/TMPRSS2 cells, and the viral titer in the culture supernatant exceeded 1.0 × 10^5^ TCID_50_/ml 5 days after infection. In addition, when the FBS concentration in the VeroE6/TMPRSS2 cell culture medium was lowered, viral replication increased in the early stages (up to 4 days after infection) but slowed down in the late stages (4 to 6 days after infection). Finally, 6 days after infection, the viral titer in the culture supernatant of VeroE6/TMPRSS2 cells cultured in the 5% FBS condition was slightly higher than that in the 2% FBS condition (*P* = 0.072) (Fig. 4).

The viral titer in the culture supernatant began to increase 3 days after infection of HCT-8 cells, and the titer exceeded 1.0 × 10^5^ TCID_50_/ml 5 days after infection. Finally, the titer in the culture supernatant 6 days after infection was almost the same for VeroE6/TMPRSS2 and HCT-8 cells (*P* = 0.678). The titer began to increase 4 days after infection of VeroE6 cells, and the viral replication in VeroE6 cells was clearly slower than that in VeroE6/TMPRSS2 and HCT-8 cells. Finally, 6 days after infection, the viral titer in the culture supernatant of VeroE6 cells was significantly lower than that of VeroE6/TMPRSS2 and HCT-8 cells (*P* = 0.016 and *P* = 0.006, respectively) (Fig. 4).

### Comparison between CPE-based and IPA-based viral titers.

**FIG 5:**
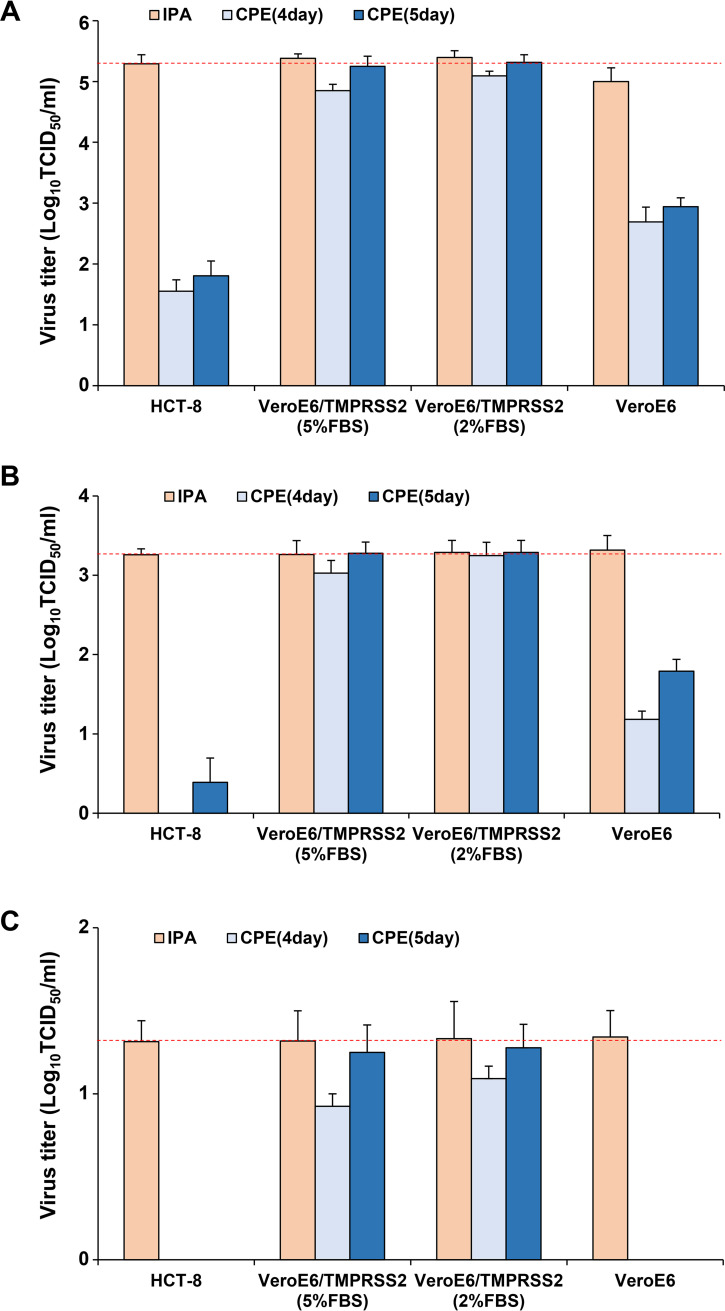
Comparison between CPE-based and IPA-based titer measurements. HCoV-OC43 sample solutions with titers of 2.0 × 10^5^, 2.0 × 10^3^, and 2.0 × 10^1^ TCID_50_/ml were prepared as high-titer, medium-titer, and low-titer virus samples, respectively. High-titer (A), medium-titer (B), and low-titer (C) HCoV-OC43 samples were titrated with CPE evaluation and the IPA method. HCT-8, VeroE6, and VeroE6/TMPRSS2 cells were used for viral titration, and VeroE6/TMPRSS2 cells were cultured in DMEM supplemented with 2% or 5% fetal bovine serum during the titration. For each measurement, four independent experiments were performed, and the results are expressed as mean ± standard error values. The reference titer value, which is the titer measured with IPA using HCT-8 cells, is indicated by the dotted red line on each panel.

The high-titer, medium-titer, and low-titer HCoV-OC43 samples were titrated with CPE evaluation and IPA. The titers measured with CPE evaluation using both HCT-8 and VeroE6 cells were considerably lower than the reference titer values. In contrast, although the titers measured with CPE evaluation 4 days after infection using VeroE6/TMPRSS2 were slightly lower than the reference titer values, the CPE-based titers 5 days after infection were almost the same as the reference titer values (Fig. 5).

Next, CPE-based titers using HCT-8, VeroE6, and VeroE6/TMPRSS2 cells were compared with the reference titer values. The CPE-based titers using HCT-8 and Vero cells had a large titer divergence, whereas the CPE-based titers using VeroE6/TMPRSS2 cells had a very small titer divergence. In particular, when the titer was measured with CPE evaluation 5 days after infection using VeroE6/TMPRSS2 cells, the 95% confidence interval of the titer divergence was in the range of −0.5 to 0.5. Under 2% FBS conditions, the 95% confidence interval of the titer divergence was in the range of −0.5 to 0.5, even when the titer was measured with CPE evaluation 4 days after infection (Table 2).

**TABLE 2:** Titer divergence of TCID_50_ assay for HCoV-OC43 with CPE evaluation using each cell line

Cell line	Titer	Titer divergence (mean [95% CI])^*b*^	
		Day 4	Day 5
HCT-8	2.0 × 10^5^^*a*^	−3.75 (−3.84 to −3.66)	−3.54 (−3.70 to −3.39)
	2.0 × 10^3^	−3.25 (−3.34 to −3.16)	−3.04 (−3.40 to −2.68)
	2.0 × 10^1^	−1.29 (−1.45 to −1.14)	−1.29 (−1.45 to −1.14)
VeroE6/TMPRSS2 (5% FBS)	2.0 × 10^5^	−0.42 (−0.76 to −0.08)	−0.04 (−0.25–0.16)
	2.0 × 10^3^	−0.29 (−0.54 to −0.04)	0.01 (−0.22–0.24)
	2.0 × 10^1^	−0.38 (−0.58 to −0.17)	−0.08 (−0.18–0.01)
VeroE6/TMPRSS2 (2% FBS)	2.0 × 10^5^	−0.17 (−0.4–0.06)	0.04 (−0.27–0.35)
	2.0 × 10^3^	−0.04 (−0.28–0.20)	0.01 (−0.12–0.14)
	2.0 × 10^1^	−0.21 (−0.45–0.04)	−0.04 (−0.32–0.24)
VeroE6	2.0 × 10^5^	−2.71 (−2.99 to −2.43)	−2.33 (−2.56 to −2.10)
	2.0 × 10^3^	−2.08 (−2.24 to −1.92)	−1.50 (−1.77 to −1.23)
	2.0 × 10^1^	−1.29 (−1.45 to −1.14)	−1.29 (−1.45 to −1.14)

a HCoV-OC43 samples with titers of 2.0 × 10^5^, 2.0 × 10^3^, and 2.0 × 10^1^ TCID_50_/ml were titrated with CPE evaluation and IPA. CPE evaluation was performed 4 and 5 days after infection.

b The titer value measured with IPA using HCT-8 cells (conventional method) was defined as the reference titer value. Next, the difference between the logarithmic value of the CPE-based titer and the logarithmic value of the reference titer was calculated, and the difference was defined as “titer divergence.” A protocol in which the 95% confidence interval of the titer measurement accuracy is completely within the range of −0.5 to 0.5 was judged to be a protocol that can measure the correct titer.

## DISCUSSION

In this study, we hypothesized that VeroE6/TMPRSS2 cells would show clear CPE not only after SARS-CoV-2 infection but also after HCoV-OC43 infection and aimed to construct a TCID_50_ assay for HCoV-OC43 based on CPE evaluation using VeroE6/TMPRSS2 cells.

CPE evaluation in each cell reconfirmed that CPE-based titer measurement using HCT-8 and VeroE6 cells was difficult because CPE is rarely observed in these cells after HCoV-OC43 infection. In contrast, VeroE6/TMPRSS2 cells showed CPE early after HCoV-OC43 infection, and even low-titer HCoV-OC43 infection showed CPE 3 to 4 days after infection. Thus, this result suggests that infected VeroE6/TMPRSS2 cells can be titrated using CPE and is consistent with previous findings that HCoV-OC43 generally uses cell-surface TMPRSS2 for cell entry and not endosomal cathepsins (19–21).

Evaluation of viral kinetics showed that HCoV-OC43 replication was slow, taking 2 to 3 days for the viral titer in the culture supernatant to start increasing, therefore making it difficult to shorten the time required for titer measurement. In addition, the viral titer in the culture supernatant of VeroE6/TMPRSS2 cells in the early stages of infection was higher than that of HCT-8 cells. This result is considered to be one of the reasons why CPE was observed in VeroE6/TMPRSS2 cells in the early stages of infection. Alternatively, because the final viral titers in the culture supernatants of VeroE6/TMPRSS2 and HCT-8 cells were almost the same, the use of either cell for culturing HCoV-OC43 was comparable. However, for isolation culture of HCoV-OC43 from clinical samples, VeroE6/TMPRSS2 cells are considered to be suitable because a successful viral culture can be confirmed by CPE evaluation (14, 15).

In the comparison between CPE-based and IPA-based (i.e., the reference titer value) titrations, the titer measured with CPE evaluation using VeroE6/TMPRSS2 cells had a significantly smaller titer divergence than those of HCT-8 and VeroE6 cells, and the 95% confidence interval of the titer divergence was in the range of −0.5 to 0.5. Thus, these findings demonstrate that TCID_50_ assays with CPE evaluation using VeroE6/TMPRSS2 cells instead of HCT-8 or VeroE6 cells can measure the correct titer value. Furthermore, the correct titer value can be measured by observing CPE at 5 days after infection and at 4 days after infection when the FBS concentration in VeroE6/TMPRSS2 cell culture medium is reduced from 5% to 2%. We speculate that culturing VeroE6/TMPRSS2 cells in FBS concentrations lower than those required by the cells may cause earlier appearance of CPE owing to reduced cell viability. If ease of observation of CPE is a priority, we recommend HCoV-OC43 titration by CPE observation 5 days after infection with FBS concentration of 5%; the detection limit under this titration condition is 1.0 × 10^1^ TCID_50_/ml.

A limitation of the virus titer measurement method described in this study is that it takes around 4 to 5 days to complete the titer measurement, similar to the conventional measurement method. However, at present, the slow replication of HCoV-OC43 makes it difficult to shorten the time required for titer measurement. Future research is required to improve the proposed method.

In conclusion, compared with the conventional viral titer measurement method with IPA, the titer measurement method based on CPE evaluation using VeroE6/TMPRSS2 cells provides the same accuracy and does not require any staining procedures using antibodies or substrates. The introduction of this titer measurement method will greatly contribute to future research on HCoV-OC43 by realizing simple, low-cost, and accurate titer measurement of this virus.

## MATERIALS AND METHODS

### Cells.

HCT-8 (ATCC CCL-244) cells were purchased from the American Type Culture Collection (Manassas, VA, USA) and were cultured in RPMI 1640 medium (FUJIFILM Wako Pure Chemical Corporation, Osaka, Japan) supplemented with 10% fetal bovine serum and standard antibiotics. VeroE6 cells were purchased from the Japanese Collection of Research Bioresources Cell Bank (Osaka, Japan) and were cultured in Eagle's minimal essential medium (MEM) (Sigma-Aldrich, St. Louis, MO, USA) supplemented with 10% fetal bovine serum and standard antibiotics. VeroE6/TMPRSS2 cells (cell number JCRB1819), expressing the transmembrane serine protease, TMPRSS2, were purchased from the Japanese Collection of Research Bioresources Cell Bank and were cultured in Dulbecco's modified Eagle's medium (DMEM) (Sigma-Aldrich) supplemented with 5% fetal bovine serum and G418 (Nacalai Tesque, Kyoto, Japan) (14, 15). The VeroE6/TMPRSS2 cells used herein were treated by the Japanese Collection of Research Bioresources Cell Bank with antimycoplasma reagents to remove the mycoplasmas and were reported to be mycoplasma-negative when monitored for 3 months. Moreover, using the EZ-PCR mycoplasma test kit (Biological Industries, Beit HaEmek, Israel), we also confirmed that the VeroE6/TMPRSS2 cells used in this study were mycoplasma free.

### Viruses.

HCoV-OC43 (ATCC VR-1558) was purchased from the American Type Culture Collection. The virus was cultured in HCT-8 cells and stored as a working stock at −80°C. The virus was concentrated and purified as follows: 120 h postinfection, the culture medium was harvested and centrifuged for 10 min at 2,500 × *g* at 4°C to eliminate the cellular debris. After centrifugation, the supernatants were sterilized by passage through a 0.22-μm filter, and virions in the supernatant were sedimented through a 20% (wt/wt) sucrose cushion in phosphate-buffered saline (PBS) with ultracentrifugation at 28,000 rpm for 2.5 h at 4°C in a Beckman SW28 rotor (17, 22).

### Viral titer measurement with IPA (conventional method).

The titer values of infectious HCoV-OC43 virions were determined with IPA as previously reported (4–9). Briefly, HCT-8 cells were seeded in 96-well plates and infected with each sample serially diluted from 1.0 × 10^0^- to 1.0 × 10^7^-fold. Cells were incubated for 4 days, and then fixed with 4% paraformaldehyde and permeabilized with 0.1% Triton X-100. A monoclonal mouse antibody specific for the HCoV-OC43 nucleoprotein (MAB9013; Sigma-Aldrich) and horseradish peroxidase (HRP)-conjugated horse anti-mouse IgG antibody (Vectastain Elite ABC mouse IgG kit; Vector, Burlingame, CA, USA) were used as the primary and secondary antibodies, respectively. Immune complexes were detected with 3,3-diaminobenzidine tetrahydrochloride (DAB substrate kit; vector) and 0.01% hydrogen peroxide in PBS, and the viral titers were calculated with the Karber method.

### Evaluation of HCoV-OC43 kinetics.

HCT-8, VeroE6, and VeroE6/TMPRSS2 cells were seeded in 12-well plates and infected with a specific titer of HCoV-OC43 (1.0 × 10^2^ TCID_50_/ml). The viral titer in the culture supernatant of each well was measured from 1 to 6 days after infection. VeroE6/TMPRSS2 cells were cultured in DMEM supplemented with 2% or 5% fetal bovine serum after virus inoculation.

### CPE evaluation and comparison between CPE-based and IPA-based titers.

HCoV-OC43 sample solutions with titers of 2.0 × 10^5^, 2.0 × 10^3^, and 2.0 × 10^1^ TCID_50_/ml were prepared and used for subsequent evaluation as high-titer, medium-titer, and low-titer virus samples, respectively.

First, HCT-8, VeroE6, and VeroE6/TMPRSS2 cells were seeded in 96-well plates and infected with high-titer, medium-titer, and low-titer HCoV-OC43 samples, and the corresponding CPEs were evaluated from 1 to 5 days after infection. CPE was observed with an inverted light microscope (Olympus IX71; Olympus, Tokyo, Japan). VeroE6/TMPRSS2 cells were cultured in DMEM supplemented with 1%, 2%, 3%, 4%, or 5% fetal bovine serum after virus inoculation.

Next, high-titer, medium-titer, and low-titer HCoV-OC43 samples were titrated with CPE observation and the IPA method. The titer measurement by CPE observation was performed as follows (14, 15, 17, 23): HCT-8 cells, VeroE6, and VeroE6/TMPRSS2 cells were seeded in 96-well plates and infected with each sample serially diluted from 1.0 × 10^0^- to 1.0 × 10^7^-fold. After the cells were incubated for 4 or 5 days, the CPE in each well was scored under a microscope, and the TCID_50_ was calculated. The titer measured with the IPA using HCT-8 cells, the conventional method, was defined as the reference titer value, and the titer values measured with CPE observation using HCT-8, Vero, and VeroE6/TMPRSS2 cells were compared with the reference values.

Four independent experiments were performed for each measurement. The titer values are expressed as means ± standard error of the mean values.

### Statistical analysis.

Titer values were analyzed using the GraphPad Prism 7 software (GraphPad, La Jolla, CA, USA). Specifically, the titer value measured with IPA using HCT-8 cells was defined as the reference titer value. Next, the difference between the logarithmic value of the CPE-based titer and the logarithmic value of the reference titer was calculated, and this difference was defined as “titer divergence.” A protocol in which the 95% confidence interval of the titer measurement accuracy is completely within the range of −0.5 to 0.5 was judged to be a protocol that can measure the correct titer.
